# Attitudes and prevention towards malaria in the context of COVID-19 pandemic in urban community in Benin, West Africa

**DOI:** 10.1186/s12936-023-04663-7

**Published:** 2023-08-04

**Authors:** Donald Hessou-Djossou, Innocent Djègbè, Yêyinou Laura Estelle Loko, Massioudou Koto Yerima Gounou Boukari, Odilon M. Nonfodji, Geneviève Tchigossou, Rousseau Djouaka, Martin Akogbeto

**Affiliations:** 1 Département des Sciences de la Vie et de la Terre, Ecole Normale Supérieure de Natitingou, UNSTIM, Natitingou, Bénin; 2 Plateforme Agriculture Environnement Santé, Institut International d’Agriculture Tropicale (IITA-Bénin), Cotonou, Bénin; 3 École Nationale Supérieure des Biosciences et Biotechnologies Appliquées (ENSBBA), Dassa, Bénin; 4 Laboratoire de Chimie de l’Eau et de l’Environnement (LCEE), Ecole Normale Supérieure de Natitingou, UNSTIM, Natitingou, Bénin; 5 Centre de Recherche Entomologique de Cotonou (CREC), Ministère de la Santé, Cotonou, Bénin

**Keywords:** COVID-19, Malaria, Attitudes, Practices, Benin

## Abstract

**Background:**

The COVID-19 pandemic and its damages have severely impacted the global healthcare system even in countries with the best systems. In sub-Saharan Africa (SSA), it could worsen the malaria situation in endemic countries such as Benin. This study was conducted to describe the potential effects of the pandemic on urban dwellers attitudes, prevention and treatment against malaria in four major cities of Benin.

**Methods:**

A cross-sectional questionnaire survey was conducted in Cotonou, Bohicon, Parakou and Natitingou, four urban cities of Benin. A total of 800 randomly selected households were interviewed. The questionnaire consisted of four parts: sociodemographic characteristics, knowledge, attitude, and practice. Descriptive statistics and binomial logistic regression analysis were used in the statistical analysis.

**Results:**

More than 90% of the participants interviewed had a good level of knowledge about the transmission and prevention of malaria in the cities surveyed. In contrast, low proportions of participants reported visiting health-care facilities when they suspected malaria. Compared to the proportions observed at Parakou and Natitingou, the low proportion of participants was statistically significant at Cotonou (Parakou: *X*^2^ = 31.345, *df* = 1, *P* < 0.0001; Natitingou: *X*^2^ = 17.471, *df* = 1, *P* < 0.0001). Among the reasons for not seeking care, these related to COVID-19 were the most mentioned. Moreover, the good education level of the participants was one of the factors associated with the non-use of healthcare facilities due to over-knowledgeable about Covid-19, which might have increased the fear to go to the health facilities. Finally, high proportions of self-medication practice were mentioned with high use of malaria drugs to treat both malaria and to protect against COVID-19.

**Conclusions:**

The data show a negative impact of COVID-19 on visits to healthcare facilities for malarial treatment and malaria drugs usage by the population. It is, therefore, necessary to rebuild malaria programmes by integrating measures adapted to health crises such as the COVID-19 pandemic.

## Background

Over the last two decades, enormous progress has been made in reducing malaria burden in Africa, following the scale-up of effective malaria control interventions [1, 2]. According to a recent World Health Organization (WHO) report, from 2000 to 2019, the incidence of malaria fell from 368 cases per 1000 inhabitants to 222.9. Moreover, the mortality rate linked to malaria fell from 149.6 cases per 1000 inhabitants to 56 [3]. These several efforts are largely attributed to the global strategy for fighting malaria by expanding the distribution of impregnated mosquito bed nets, indoor spraying of residual insecticides, other vector control strategies and more effective anti-malarial treatments [4, 5].

Despite these progresses, malaria transmission persists in African urban areas and in some cases, at even higher levels than in surrounding areas [6]. It is estimated that 24.8–103.2 million clinical malaria episodes occur annually in urban settings where malaria is endemic [7]. Some factors, including uncontrolled urban expansion, large-scale practice of urban agriculture and the increasing number of people moving to urban areas, may contribute to this and probably affect the dynamics and epidemiology of malaria [8].

With the outbreak of the COVID-19 pandemic, regulations or restriction measures implemented by different governments to curtail virus spread may have major repercussions on malaria treatment and prevention, including decreased access to health care, interruption of service delivery, and disruption of delivery of malaria control interventions [2]. These direct and indirect effects of the COVID-19 pandemic would worsen the malaria situation, particularly in urban areas of endemic countries. Indeed, 90% of global reported COVID-19 cases occurred in urban communities and lockdown measures there were more accentuated [9].

According to Weiss et al. [1], the COVID-19 lockdown and its disruptions in malarial intervention could almost double malaria mortality in 2020 and potentially lead to higher mortality rates in subsequent years in Africa. Although the data recorded at the end of 2020 are quite far from predicted analysis, an increase of the total number of malaria cases has nevertheless been recorded, rising from 213 million to 228 million [10]. Moreover, a study conducted in South Africa showed that lockdowns were associated with a large and significant decline in the use of health services among children [11]. In the Democratic Republic of Congo (DRC), a drop in attendance at health facilities for malaria treatment was reported, depending on local lockdown measures [12]. Another study carried out in Uganda found that the onset of the COVID-19 pandemic did not have major effects on malaria disease burden or indicators of case management [2].

In Benin, Aïkpon et al. [13] reported many challenges for the implementation of the 2020 ITNs mass distribution campaign due to the COVID-19 pandemic. This campaign owes its success to the revision of the initial distribution protocol and strong support from the Government, through the Ministry of Health, to continue with the implementation of the ITNs campaign in advance of the high transmission malaria season, during and despite the COVID-19 pandemic. Apart from this report, no other studies have been conducted to determine how the pandemic has effectively impacted the malaria burden and control, or the health service delivery and their use by the populations. This information is important to design better program interventions adapted to epidemic situations such as COVID-19 and to mitigate its negative impacts. This will help to sustain progress made on malaria intervention coverage in the future. The current study contributes to address this gap by assessing dwellers attitudes and practices towards malaria prevention and treatment in four urban cities, during the COVID-19 pandemic in Benin.

## Methods

### Study area

**Fig. 1:**
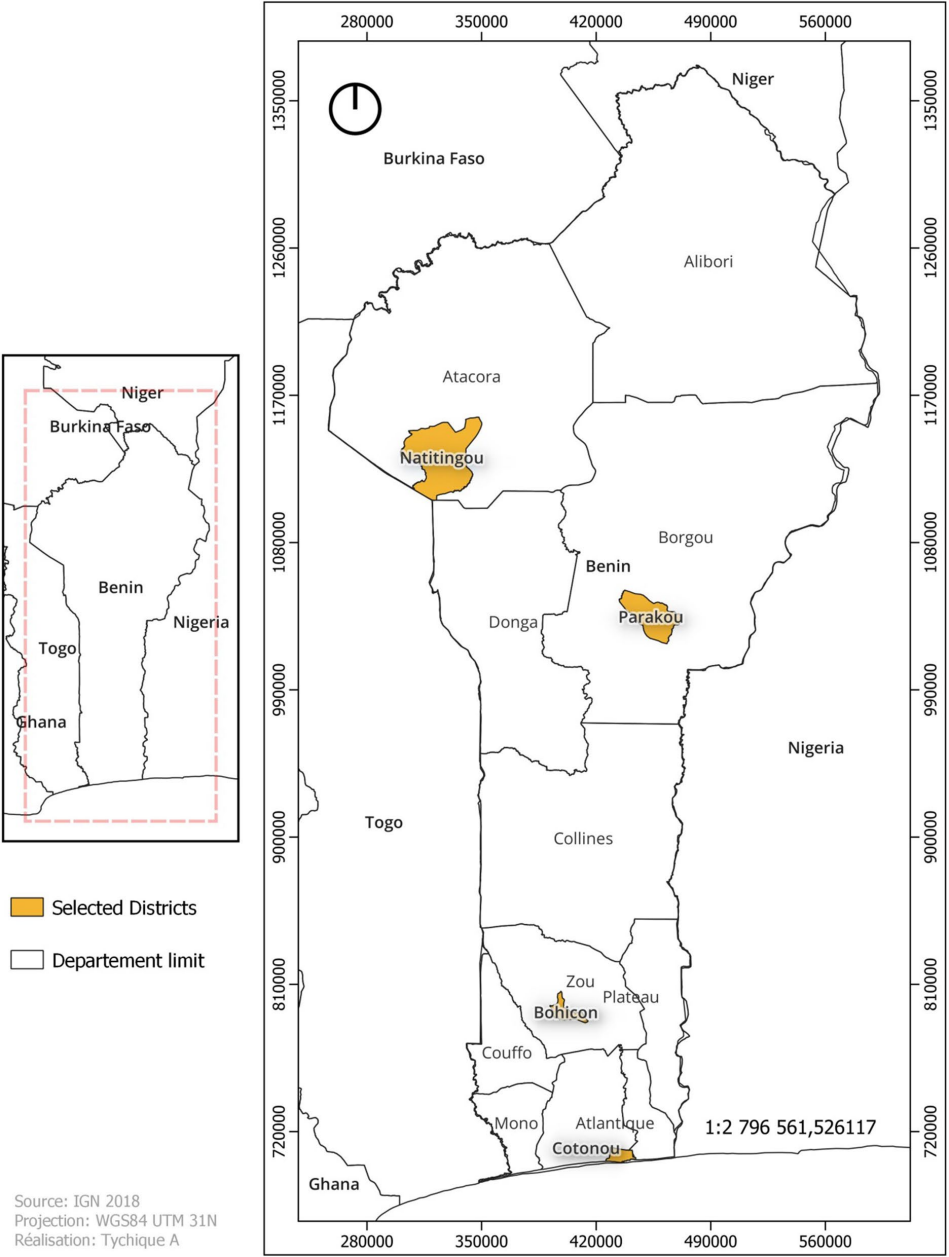
Benin map highlighting study sites

Surveys were conducted in four major urban cities of Benin: Cotonou (6°21′55.3″ North, 2°25′6″ East), Bohicon (7°10′41.7″ North, 2°4′0.1″ East), Parakou (9°20′13.8″ North, 2°37′49.1″ East), and Natitingou (10°18′15″ North, 1°22′46.6″ East) (Fig. 1). The choice of these cities was justified by the fact that they are of the most important cities in the country in terms of population density, making them the epidemiological foci of the COVID-19. They are also characterized by a large mobility of the population and the development of several sectors of activities, such as tourism, trade and industrial manufacturing activities, which are sources of economic income.

Cotonou, the economic capital of Benin is situated in the department of Littoral on the coastal strip that stretches between Nokoué Lake and the Atlantic Ocean. It is the largest town, main port of Benin, and covers an area of 79 km^2^ with a population of 1,228,667 inhabitants [14]. The city is full of a large number of processing and storage factories [15].

Bohicon is situated in central Benin and covers an area of 44 km^2^ with a population of 171,781 inhabitants [14]. The main activities of the population are trade, crafts and urban agriculture [16].

Parakou is in the northeastern part of Benin. It covers an area of 441 km^2^ with a population of 254,254 inhabitants [14]. Trade and urban agriculture are the main activities in this town [17].

Natitingou is located in the northwestern Benin, and covers an area of ​​3045 km^2^ with a population of 103,843 inhabitants [14]. The main activities are agriculture, tourism, crafts and trade [18].

### Sampling

The minimum sample size for the study was calculated using the single population proportion formula (n = (Zα/2)^2^p((1 − p)/d^2^)) at a 95% confidence interval (CI) (Zα/2 = 1.96), with 5% margin of error [19]. Using P to be 44.9% from the WHO Benin report [20], the sample size required for conducting this study was 380 households. For better representativeness of the population, we surveyed 200 participants per city, i.e. a total of 800 in all four cities.

### Surveys

The present study was a community-based descriptive cross-sectional study, with the aim of assessing population knowledge and attitudes about malaria prevention and treatment to adapt to restrictive health measures during the COVID-19 period in urban cities of Benin.

Before the beginning of the survey, interviewers were trained on how to use the questionnaire and on methods to approach respondents and obtain consent. The first confirmed COVID-19 case was seen in Benin on March 16, 2020 and the survey was conducted five months later, from August to September 2020. At each study site, the households were randomly selected and the person who consented to participate in the study (household head, spouse or and elder representative of the house) was interviewed. Interviews were undertaken in French or the local language spoken in each city and in private to reduce influence from other people. Data collected include people’s knowledge about malaria signs, knowledge and attitude on preventive measures; and attitudes in case of malaria suspicion during the period of restrictive healthy measures linked to COVID-19. Some demographic variables such as the age, gender, level of education, profession of the respondent, household composition and house appearance were also recorded.

### Data analysis

The collected data were analysed using the SPSS 24.0 statistical software package. Means and proportions were used for descriptive analysis. Percentages were compared using the chi-squared test. Comparisons between means were assessed using ANOVA.

## Results

### Sociodemographic characteristics of surveyed households

In the four cities, 59.3% (n = 474) of the interviewed participants were females and 40.7% (n = 426) were males. The majority of the participants were 25–34 years old (33.3%) with the average age of 37.0 ± 12.3. The post primary level was the highest level of education attained by the majority of participants (41%). Among them, the housewives accounted for the largest group in this study (33.6%) followed by the traders (29.3%). Family sizes are large with the majority (49.5%) reporting having between six and ten (6–10) household members (Table 1).

**Table 1:** Sociodemographic characteristics of households surveyed

Items Characteristics	Parakou n (%)	Natitingou n (%)	Bohicon n (%)	Cotonou n (%)	Total n (%)
Age					
18–24	29 (14.5)	36 (18)	51 (25.5)	17 (8.5)	133 (16.6)
25–34	94 (47)	78 (39)	66 (33)	28 (14)	266 (33.3)
35–44	52 (26)	41 20.5)	43 (21.5)	58 (29)	194 (24.2)
45–54	16 (08)	20 (10)	37 (18.5)	41 (20.5)	114 (14.3)
55 and above	09 (4.5)	25 (12.5)	3 (1.5)	56 (28)	93 (11.6)
Gender					
Male	42 (21)	94 (47)	112 (56)	78 (39)	326 (40.7)
Female	158 (79)	106 (53)	88 (44)	122 (61)	474 (59.3)
Education					
No formal schooling	58 (29)	50 (25)	64 (32)	20 (10)	192 (24)
Primary level	84 (42)	78 (39)	46 (23)	72 (36)	280 (35)
Post primary level	58 (29)	72 (36)	90 (45)	108 (54)	328 (41)
Appearance of dwellings					
Grouped	126 (63)	2 (01)	0	46 (23)	174 (21.7)
Ungrouped	74 (37)	198 (99)	200 (100)	154 (77)	626 (78.3)
Number of people in households					
1–5	81 (40.5)	95 (46.5)	61 (30.5)	103 (51.5)	340 (42.5)
6–10	111 (55.5)	103 (51.5)	117 (58.5)	65 (32.5)	396 (49.5)
> 10	8 (04)	2 (01)	22 (11)	32 (16)	64 (8)
Occupational status					
Manual workers	12 (06)	72 (46)	39 (19.5)	84 (42)	207 (25.8)
White-collar workers	07 (3.5)	18 (09)	06 (03)	22 (11)	53 (6.7)
House wife	73 (36.5)	99 (49.5)	61 (30.5)	36 (18)	269 (33.6)
Traders	99 (49.5)	11 (5.5)	74 (37)	50 (25)	234 (29.3)
Students	2 (01)	0	10 (05)	01 (0.5)	13 (1.6)
Others	7 (3.5)	0	10 (05)	07 (3.5)	24 (3)

### Knowledge and attitude of household respondents towards malaria

Almost all the interviewed participants (98.5%) in the four cities knew that malaria is transmitted by the mosquito bites. However, some participants associated malaria with lack of hygiene, for others it was caused by the sun or even the consumption of certain foods (Table 2).

**Table 2:** Knowledge about malaria and prevention methods in the four cities

Characteristics	Parakou n (%)	Natitingou n (%)	Bohicon n (%)	Cotonou n (%)	Total N (%)
Means of transmission					
Mosquito bites	192 (96)	198 (99)	198 (99)	200 (100)	788 (98.5)
Lack of hygiene	71 (35.5)	26 (13)	31 (15.5)	15 (7.5)	143 (17.9)
Sun	2 (01)	0	28 (14)	11 (5.5)	41 (5.1)
Some foods	0	1 (0.5)	3 (1.5)	1 (0.5)	5 (0.6)
Witchcraft	0	0	2 (01)	1 (0.5)	3 (0.4)
Symptom of malaria					
Fever	200 (100)	200 (100)	200 (100)	199 (99.5)	799 (99.9)
Headache	123 (61.5)	112 (66)	177 (88.5)	148 (74)	560 (70)
Chill	182 (91)	173 (86.5)	180 (90)	136 (68)	671 (83.9)
Loss of appetite	87 (43.5)	61 (30.5)	122 (61)	109 (54.5)	379 (47.4)
Joint pain	54 (27)	18 (9)	38 (19)	73 (46.5)	183 (22.9)
Vomiting	26 (13)	39 (19.5)	27 (15.5)	48 (24)	140 (17.5)
Anaemia	08 (4)	02 (1)	33 (16.5)	12 (6)	55 (6.9)
Others	00	02 (1)	00	01 (0.5)	3 (0.4)
Mosquito protection					
LLINs	160 (80)	182 (91)	189 (94.5)	198 (99)	729 (91.1)
Ordinary nets	14 (07)	06 (3)	13 (6.5)	08 (4)	41 (5.1)
Insecticide spray	42 (21)	24 (12)	08 (4)	08 (4)	82 (10.2)
Smoke coil	92 (46)	76 (38)	68 (34)	112 (66)	348 (43.5)
Electric hob killer mosquito	4 (02)	0	0	7 (3.5)	11 (1.4)
Window screens	38 (19)	12 (6)	10 (5)	31 (15.5)	91 (11.4)
Blanket	0	0	15 (7.5)	24 (12)	39 (4.9)
Sniper	3 (1.5)	0	1 (0.5)	5 (2.5)	9 (1.1)
Local plants	9 (4.5)	13 (6.5)	5 (2.5)	0	27 (3.4)

Regarding malaria symptoms, “fever” was mentioned, as the first by all the respondents. The answers also included chill and headache which were cited by over 65% of participants at each study site. Other symptoms mentioned were “loss of appetite” (Parakou: 43.5%; Natitingou: 30.5%; Bohicon: 61%; Cotonou: 54.5%), “joint pain” (Parakou: 27%; Natitingou: 09%; Bohicon: 19%; Cotonou: 46.5%), “vomiting” (Parakou: 13%; Natitingou: 19.5%; Bohicon: 15.5%; Cotonou: 24%), and “anaemia” (Parakou: 4%; Natitingou: 1%; Bohicon: 16.5%; Cotonou: 6%).

In all four cities, a high number of the respondents reported using impregnated mosquito bed nets as the main way of preventing malaria. The other mean prevention measures most cited were smoke coil, insecticide spray and window nets (Table 2).

Out of the 800 interviewed households, only 184 (23%) reported going systematically to health centres when they felt malaria symptoms. The proportions of those who said they went to the hospital were 34.5% at Parakou, 27.5% at Natitingou, 19% at Bohicon, and 11% at Cotonou. Significant differences were observed between these proportions at Cotonou compared to Parakou (*X*^2^ = 31.345, *df* = 1, *P* < 0.0001) and Natitingou (*X*^2^ = 17.471, *df* = 1, *P* < 0.0001).

Concerning the reasons for non-resort to health care, the majority of households attributed this attitude to self-medication and COVID-19 pandemic. Among COVID reasons, fear of contracting COVID-19 (62.8%), fear of testing positive (26.4%) or the pandemic restrictions (54.9%) were the most reported in all the four cities. Comparisons of these proportions between Cotonou and the other cities showed a significant difference with Parakou (*X*^2^ = 11.266, *df* = 1, *P = 0.0008*), Natitingou (*X*^2^ = 7.225, *df* = 1, *P = 0.0072*) and Bohicon (*X*^2^ = 78.924, *df* = 1, *P* < 0.0001).

The majority of participants in the four study sites declared receiving appropriate drugs in pharmacies (Parakou: 57%, Natitingou: 78.5%, Bohicon: 69.5%, Cotonou: 82%). The use of traditional medicines was also cited by the household representatives (Table 3). Moreover, some interviewed participants (14.9%) declared systematically following the COVID-19 treatment protocol established by health authorities, when they felt malaria symptoms.

**Table 3:** Home management of suspicion malaria cases and reasons in households

Items Characteristics	Parakou n (%)	Natitingou n (%)	Bohicon n (%)	Cotonou n (%)	Total N (%)
Systematic resort to facility care					
Yes	69 (34.5)	55 (27.5)	38 (19)	22 (11)	184 (23)
No	131 (65.5)	145 (72.5)	162 (81)	178 (89)	616 (77)
Reasons of no resort to facility care					
Fear of getting COVID-19	91 (69.5)	73 (50.3)	108 (66.7)	115 (64.6)	387 (62.8)
Fear to be tested COVID-19 positive	22 (16.8)	11 (7.6)	36 (22.2)	94 (52.8)	163 (26.4)
COVID-19 restriction measure	74 (56.5)	88 (60.7)	43 (26.5)	133 (74.7)	338 (54.9)
Self-medication	84 (64.1)	117 (80.7)	137 (84.5)	162 (91.5)	500 (81.2)
Lack of financial means	78 (59.5)	62 (42.7)	75 (46.3)	46 (25.8)	261 (42.3)
Traditional care use	36 (27.4)	28 (19.3)	74 (45.6)	15 (8.4)	153 (24.8)
Malaria treatment					
Pharmacy	114 (57)	157 (78.5)	139 (69.5)	164 (82)	574 (71.7)
Street drugs	21 (10.5)	13 (6.5)	19 (9.5)	07 (3.5)	60 (7.5)
Traditional	75 (37.5)	30 (15)	86 (43)	64 (32)	255 (31.9)
COVID-19 treatment	18 (9)	0	09 (4.5)	92 (46)	119 (14.9)

### Educational level and malaria treatment in the COVID-19 pandemic period

Comparisons of the proportion of participants according their education level and their home management of suspected malaria cases were carried out. The results showed that the majority of participants who mentioned the COVID-19 as a reason to not resort to facility care had a more advanced level of education than the primary level with a significant difference in their proportion compared to other levels of education (X^2^ = 113.7, df = 2, p-value < 0.001) (Table 4).

**Table 4:** Association between education level of participants and their management of suspicion malaria cases

Highest level of education completed	Systematic resort to facility care	Non resort to facility care	
		COVID-19 linked reasons	Others reasons
Never	63 (34.2)	102 (20.9)	139 (27.8)
Primary level	68 (37.0)	134 (27.6)	185 (37)
Post primary level	53 (28.8)	251 (51.5)	176 (35.2)
Total	184 (100)	487 (100)	500 (100)
X^2^ (p-value)	2.853 (p = 0.2401)	113.7 (p < 0.001)	10.698 (p = 0.0047)

## Discussion

The results of the present survey showed overall high proportions of participants with a good knowledge regarding malaria, its mode of transmission and prevention mechanisms. High levels of knowledge about malaria have also been reported in populations from other sub-Sahara African countries such as Cameroon, Nigeria and Burkina Faso [21–24]. This may be because of the higher level of education of respondents and socioeconomic household status [25]. Another contributing factor to this good level of knowledge is the easy access to information via different audio-visual platforms such as television, radio and social media, some of which continue to transmit health education messages on malaria [22]. However, some misconceptions still exist among respondents, and must be corrected by accentuating health communication and educational activities [26, 27].

The use of LLIN is one of the most adopted prevention methods against mosquito bites by households in the four cities. This would be due to the success of the regular LLIN mass distribution campaigns and particularly the last campaign carried out just prior to this survey and where distribution coverage of 94.16% was reached in the country [13].

Despite the good educational level noted in urban areas of the country, a low rate of utilization of health care facilities by the population was observed. The proportions recorded were much lower than WHO data, which estimated the national average for resorting to health services at 44% [28]. According to the surveyed population, the COVID-19 pandemic was the main reason for this decrease in resorting to facilities care even when they felt the symptoms of malaria. They mentioned fear of contracting the virus and pandemic restriction measures including closures of health facilities because of reduced health care workers’ capacity due to lack of personal protective equipment and stay-at-home advice by the authorities for febrile diseases. A recent study conducted in Nigeria also showed a decline in visits to health providers during the pandemic with the major reason given was the closure of health facilities [29]. According to some researchers, reduced access to health care services due to the effects of the pandemic has a negative impact on access to anti-malarial treatment thus it would likely have a major effect on the malaria burden in endemic countries such as Benin [30, 31].

In Cotonou, the higher proportion of respondents who did not go to health facilities was mainly because it was the most urban and exposed city in the country with the highest number of COVID-positive cases recorded. For this reason, after the first cases of COVID-19, Cotonou was included in the established sanitary cordon, by the government to limit the virus spread throughout the country [32]. The health measures against the pandemic were more restrictive and the population was more distressed. Stay-at-home advice, especially at the beginning of the pandemic, enhanced this bad attitude in the correct management of malaria [33].

The present study indicated that lack of financial means was an important reason to not resort to health facilities by the population during the COVID-19 pandemic. This represented an indirect consequence linked to the COVID-19 due to the inability to perform daily work by a large part of the population during lockdowns and subsequently reduced purchasing power. With the suppression of street drug trade since 2017, which was more accessible to the low income population in Benin, and the sale regulation of some malaria drugs such as chloroquine for COVID-19 treatment usage, the socioeconomic effects of COVID-19 would also increase the burden of malaria in urban areas.

Furthermore, it was found that high proportions of respondents were reported to have done self-medication. Apart from pandemic reasons, this practice is reinforced by the fact that the urban population considers itself quite informed about malaria and its treatment. Additionally in the context of self-medication, some participants revealed that in cases of suspected malaria, they systematically followed the COVID-19 treatment protocol proposed by the health authorities. This would be due first to the good knowledge level of the population about signs of the two diseases and the significant similarities between clinical manifestations of COVID-19 and malaria [34, 35]. Moreover, similar to many countries, anti-malarials were mainly used for COVID-19 prevention and treatment in Benin, thus enabling populations to prevent and fight against both COVID-19 and malaria [36]. Although this practice could have favorable effects on the malaria prevalence and incidence [30, 37], the widespread and uncontrolled use of anti-malarials to prevent and treat COVID-19 may further influence *Plasmodium* resistance in the country [38].

## Conclusions

In this study, a high level of knowledge about malaria was observed in the populations of Cotonou, Bohicon, Parakou and Natitingou. However, some misconceptions remain. In addition, the COVID-19 pandemic, through its direct and indirect consequences, has impacted the population attitudes about malaria prevention and treatment, and will likely increase the malaria burden. At the same time, the sociohealth context has led to excessive use of anti-malarial drugs for COVID-19 prevention and treatment, which may increase *Plasmodium* drug resistance and seriously hamper malaria elimination efforts in the country.

## Data Availability

The datasets used and/or analysed during the current study are available from the corresponding author on reasonable request.
